# Placement of stent grafts with different calibers into the true and false lumens of anastomotic aneurysms after descending aortic replacement for aortic dissection

**DOI:** 10.1186/s13019-023-02477-x

**Published:** 2024-01-06

**Authors:** Hiroo Uehara, Masateru Uchiyama, Tomohiro Imazuru

**Affiliations:** Department of Cardiovascular Surgery, Teikyo University Hospital, 2-11-1 Kaga, Itabashi-ku, Tokyo, 173-8605 Japan

**Keywords:** TEVAR, Modified kissing stent technique, Aortic dissection, Anastomotic aneurysms

## Abstract

Anastomotic aneurysms present as a life-threatening emergency after descending aortic replacement for aortic dissection. Thoracic endovascular aneurysm repair (TEVAR) has been performed since the early 2000s for complicated cases in which re-thoracotomy cannot be adopted. We report the case of a 57-year-old male patient, during a 5-year follow-up after descending aortic replacement for aortic dissection, developed aneurysm expansion around the false lumen on the peripheral side of the artificial graft. Considering the risk and the patient’s desires, we opted to perform TEVAR with different calibers into the true and false lumens “modified kissing stents technique”. His postoperative course was uneventful without any complications. This case highlights the utility of the modified kissing stents technique for anastomotic aneurysms after descending aortic replacement for aortic dissection using stent grafts with different calibers into the true and false lumens.

A 57-year-old male patient developed Stanford type B aortic dissection with entry on the distal side of the left subclavian artery. During a follow-up, the diameter of aortic aneurysm from the entry to Th9-10 expanded to 60 mm. We adopted a policy that descending aortic replacement (Triplex graft 22 mm, Terumo Corporation, Tokyo, Japan) from just below the left subclavian artery to Th9-10 should be performed because the length of the proximal landing zone in TEVAR would run short. During a 5-year follow-up after descending aortic replacement for aortic dissection, developed aneurysm expansion around the false lumen on the peripheral side of the artificial graft. A computed tomography (CT) and angiography revealed an expanded aneurysm with the saccular aneurysm (61 mm) around the false lumen on the peripheral side of the artificial graft anastomosed with a double-barrel technique (Fig. 1A–C). The diameters of the true and false lumen were 11 and 38 mm, respectively. Regarding the kinds of surgical interventions, we considered performing TEVAR only in the true lumen, direct coil embolization with a plug above the right renal artery, and fenestration above the right artery. However, there were some problems such as the possibility of re-entry between the pseudoaneurysm and the embolization, the cost of coils, and the possibility of leg ischemia by changes in blood flow induced by fenestration. We adopted a policy that thoracic endovascular aortic repair (TEVAR) with different calibers into the true and false lumens should be performed to perfuse both the true and false lumens considering the difference in lumen sizes, the conditions of abdominal branch vessels (Fig. 1D–H), and the patient’s desires.

**Fig. 1:**
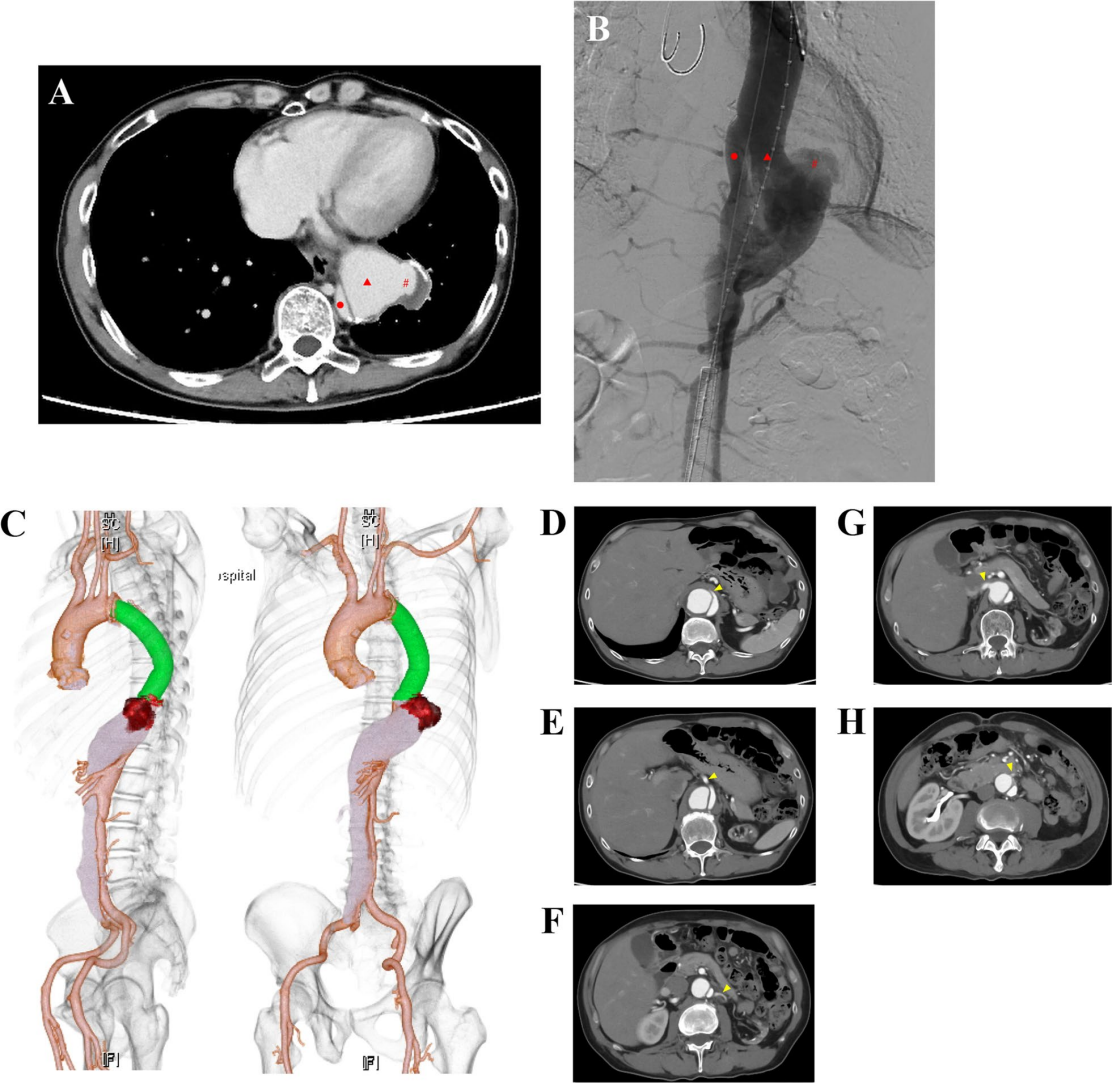
Preoperative findings. **A** Contrast-enhanced computed tomography (CT) scan. **B** Angiography of an expanded aneurysm with the saccular aneurysm. Each marker (●, ▲, and #) shows the same. **C** Three-dimensional-CT. **D**–**H** Celiac artery (**D**), superior mesenteric artery (**E**), and left renal artery **F** from the true lumen. Right renal artery (**G**) and inferior mesenteric artery **H** from the false lumen. Each artery is shown as a yellow triangle

The procedure was performed under general anesthesia. Both common femoral arteries were exposed. After exposing both common femoral arteries (CFAs), wires were inserted through the left CFA into the true lumen and through the right CFA into the false lumen, while checking with intravascular ultrasound. Excluder contralateral leg of 16–115 mm (W.L. Gore & Associates Inc, Flagstaff, Ariz) and Conformable Gore TAG (CTAG) device of 34–100 mm were placed on the true and false lumens, respectively. An additional Excluder contralateral leg of 18–135 mm in the true lumen and an additional CTAG of 37–100 mm in the false lumen were added just above the celiac artery (Fig. 2A). A balloon touch-up was simultaneously performed. Nevertheless, an Excluder aorta extender of 36–45 mm was additionally placed because type 1b endoleak around the false lumen was observed. His postoperative course was uneventful without any serious perioperative complications. Good flow in both lumens, no endoleak, and good stent-graft positioning were observed on the CT during the follow-up after three months (Fig. 2B). The patient's course remained uneventful at seven months after surgery.

**Fig. 2:**
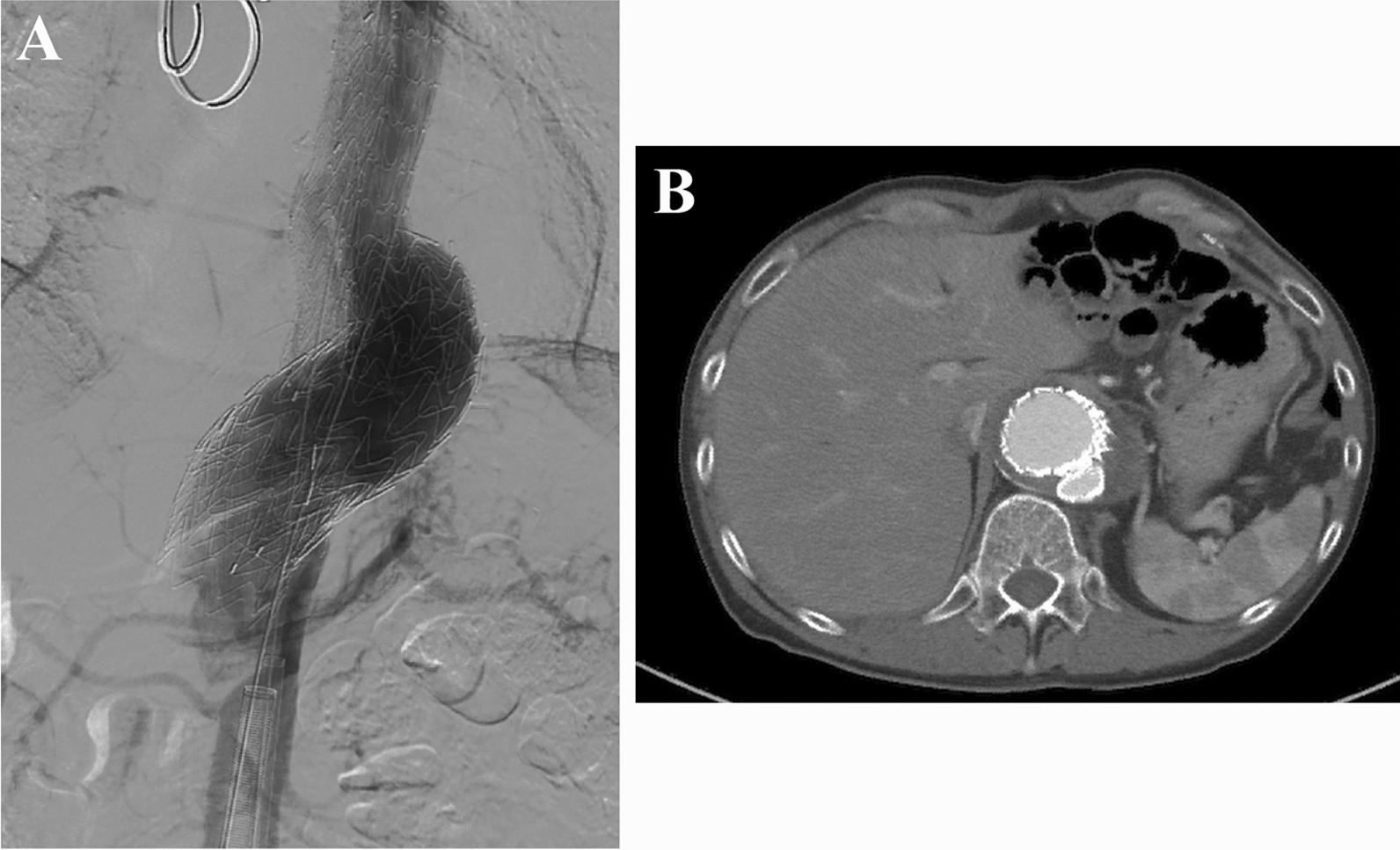
Intra- and postoperative findings. **A** Intraoperative findings under digital subtraction angiography. **B** Postoperative contrast-enhanced computed tomography scan

The use of TEVAR in patients with chronic type B aortic dissection, however, remains controversial. As in our case, one study reported that the kissing stent technique for chronic aortic dissection completely preserved blood flow to abdominal branch vessels and excluded the aneurysm by implanting the stent graft landing on the false and true lumen [1] Indeed, our case with a significant difference in vessel diameter between the true and false lumen was challenging for performing TEVAR only on the true lumen. In contrast to the previous methods of inserting stent grafts of the same diameter, our modified kissing stent technique, insertion of stent grafts with different diameters, was performed. In particular, we tried to carefully perform simultaneous touch-up to prevent the small stent graft from crushing during implantation.

This case provides valuable insight into the modified kissing stents technique using stent grafts with different calibers for aortic dissection, highlighting the importance of appropriate surgical intervention as one of the alternative treatments for patients with difficult surgical situations or refusal of open surgery.

## Data Availability

The datasets used are available from the corresponding author upon reasonable request.

## Abbreviations

CT: Computed tomography
TEVAR: Thoracic endovascular aortic repair
CFA: Common femoral artery
CTAG: Conformable Gore TAG
